# Synchronous Pelvic Schwannoma With Metastatic Prostate Cancer: A Rare Case and Pathology Review

**DOI:** 10.7759/cureus.52356

**Published:** 2024-01-16

**Authors:** Hagar Attia, John O Agboola, Gyuhee Seong, Aye Thida, Edwin Chiu, Maksim Agaronov

**Affiliations:** 1 Pathology and Laboratory Medicine, Kings County Hospital Center, New York, USA; 2 Pathology and Laboratory Medicine, State University of New York (SUNY) Downstate Health Sciences University, New York, USA; 3 Medicine, State University of New York (SUNY) Downstate Health Sciences University, New York, USA; 4 Hematology and Oncology, State University of New York (SUNY) Downstate Health Sciences University, New York, USA

**Keywords:** immunohistochemistry (ihc), oncology, surgical pathology, prostate adenocarcinoma, pelvic schwannoma

## Abstract

Schwannomas are benign tumors arising from well-differentiated Schwann cells of peripheral nerves. They are usually found on the limbs, head, and neck. It is uncommon for schwannoma to occur in the pelvis and when it does, it is often diagnosed late. Pelvic schwannoma when diagnosed are often bigger in size (>5 cm) and may present with local symptoms such as constipation and bladder outlet obstruction. We hereby present a patient with concurrent metastatic prostate carcinoma and pelvic schwannoma. The patient is a 57-year-old man initially diagnosed with prostate cancer and was lost to follow-up. One year later, he presented with metastatic prostate disease and bladder outlet obstruction. Further evaluation revealed a concurrent pelvic mass that was increasing in size. The biopsy of this mass was suggestive of schwannoma. It was decided at the multidisciplinary tumor board conference to offer treatment for his metastatic prostate disease and observe the schwannoma. His obstructive symptoms worsened in the face of clinical evidence of regression of his prostatic disease, and it was decided to resect the pelvic mass. The surgery revealed a huge soft tissue mass within the pelvis that was adherent to the bladder, prostate, and rectum. Morphology and immunohistochemistry studies of the pelvic mass confirmed the diagnosis of ancient schwannoma. We hereby highlight the clinical importance of this presentation and the diagnostic and therapeutic dilemma involved in the management of this patient who presented with two pathologic conditions causing similar symptoms but of different prognostic and therapeutic significance.

## Introduction

Pelvic schwannomas are extremely rare, benign tumors originating from well-differentiated Schwann cells of peripheral nerves. Schwannomas usually develop in the head and neck region, mediastinum, or upper and lower extremities and rarely in the pelvis (less than 0.5% without association with neurofibromatosis type 1) [1]. These tumors commonly remain asymptomatic until they attain significant size, leading to compression of adjacent organs and subsequent clinical manifestations. Patients may experience pelvic or lower back pain, urinary urgency or incontinence, and constipation as pelvic schwannomas enlarge [2]. 

Surgery is the first line of treatment for pelvic schwannoma. Due to its rarity, careful pathological and radiological evaluations are warranted. There is currently no case report with synchronous prostate cancer and pelvic schwannoma published in PubMed. We present the first case of pelvic schwannoma synchronous with stage 4 prostate cancer. 

## Case presentation

A 57-year-old male with a history of hypertension was initially diagnosed with prostate cancer at an outside hospital. Bone scan and computed tomography (CT) of the chest, abdomen, and pelvis in outside records showed no evidence of bone disease but revealed an 11 x 6.6 cm presacral mass compressing the rectum and a 0.6 x 0.5 cm right middle lobe pulmonary nodule. The patient opted for observation but was lost to follow-up for a year due to social circumstances. 

After one year, the patient presented to our institution's primary care practice with complaints of epigastric pain and constipation lasting for three months. A subsequent prostatic specific antigen (PSA) measurement revealed a significantly high level of 4360 ng/ml, leading to a referral to the Urology department. Two months later, when the patient was evaluated by Urology, the PSA level had further increased to 9957 ng/ml. A CT scan of the abdomen and pelvis showed a markedly enlarged, heterogeneous, and infiltrative prostate gland, which infiltrated the seminal vesicles and urinary bladder compatible with advanced prostate carcinoma. The patient was initiated on androgen deprivation therapy (ADT) with bicalutamide and leuprolide. 

**Figure 1:**
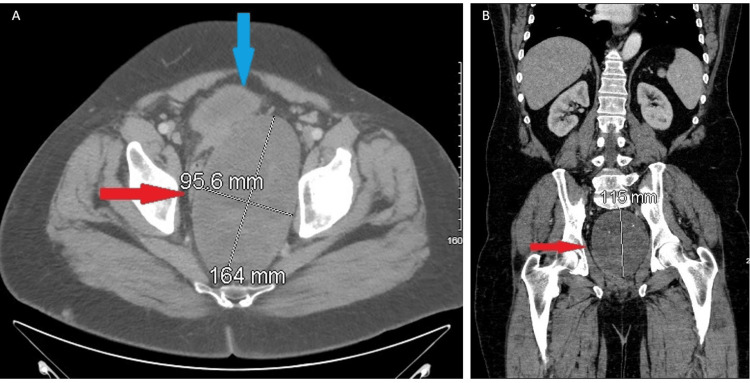
(A-B) CT abdomen/pelvis with contrast. Complex, heterogeneous, encapsulated solid mass (red arrow) within the pelvis measuring up to 16.4 cm. Markedly enlarged, heterogeneous, and infiltrative prostate gland (blue arrow), which obliterates the seminal vesicles and invades the urinary bladder in keeping with advanced prostate carcinoma.

Further radiological evaluation with CT abdomen/pelvis with contrast revealed peritoneal carcinomatosis and a complex pelvic mass measuring 15 x 10 cm causing deviation of the rectum and bladder (Figure 1). These findings were atypical for metastatic prostate cancer. Bulky iliac chain lymphadenopathy was also noted. A bone scan showed osseous metastases to the right iliac bone and femoral head.

**Figure 2:**
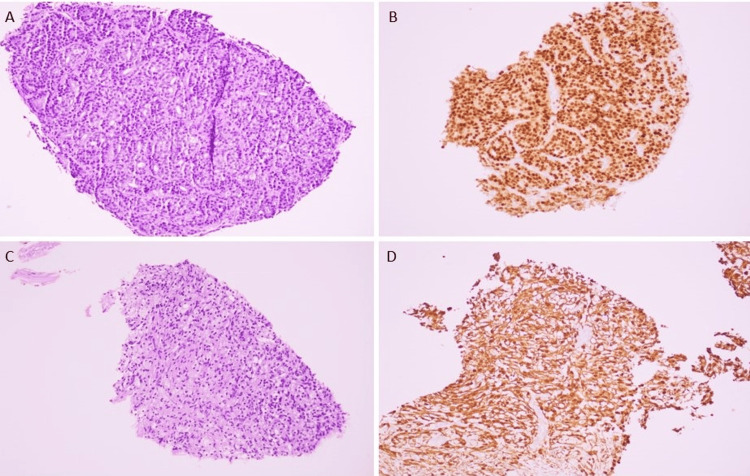
(A) Hematoxylin and eosin (H&E) (200X magnification) of the abdominal soft tissue biopsy showing poorly formed glands. (B) The glands are positive for NKX3.1 consistent with metastatic prostate adenocarcinoma. (C) H&E (200X magnification) of the pelvic mass biopsy consists of bland-appearing spindle cells. (D) The spindle cells are positive for S100.

Subsequently, the patient was referred to Interventional Radiology for a biopsy of the pelvic mass and the soft tissue of the abdomen. The biopsy of the abdominal soft tissue was positive for metastatic prostate adenocarcinoma (Figure 2A-2B). The pelvic mass was determined to be a peripheral nerve sheath tumor, such as schwannoma. The tumor exhibited spindle cells and tested positive for S100 and SOX10 while being negative for CD45, CD68, and HMB45 (Figure 2C-2D). He initially had a good PSA response, which decreased to 100 ng/ml. However, in one month, PSA increased to 800 ng/ml, with testosterone being 10 ng/dL. The patient underwent a laparoscopic biopsy of peritoneal carcinomatosis, which was deemed to be metastatic prostate cancer.

This case was discussed in the multi-disciplinary tumor board conference, and the consensus was to give prostate-directed therapy with chemo-hormonal therapy. The patient was started on docetaxel/prednisone with androgen deprivation therapy (Leuprolide) and completed six cycles. Follow-up CT scans showed a persistently enlarged prostate without lymphadenopathy or peritoneal carcinomatosis and increased scattered sclerotic lesions. Bone scan demonstrated persistent uptake in the right iliac bone, femoral head, and scattered vertebral bodies compatible with osseous metastasis. Subsequent PSA was found to be elevated to 182 ng/ml from 58 ng/ml from the prior visit. Thus, the patient was confirmed to have metastatic castrate-resistant prostate cancer. Given the progression of bone lesions and poor biochemical response to chemotherapy, the patient was transitioned to the second line of abiraterone/prednisone with androgen deprivation therapy (leuprolide). 

Next generation sequencing panel was normal with stable microsatellite instability (MSI), low tumor mutation burden, and without ARV7 or DNA repair defect mutation. There was a genetic variant of unknown significance APC gene p.P441A. 

**Figure 3:**
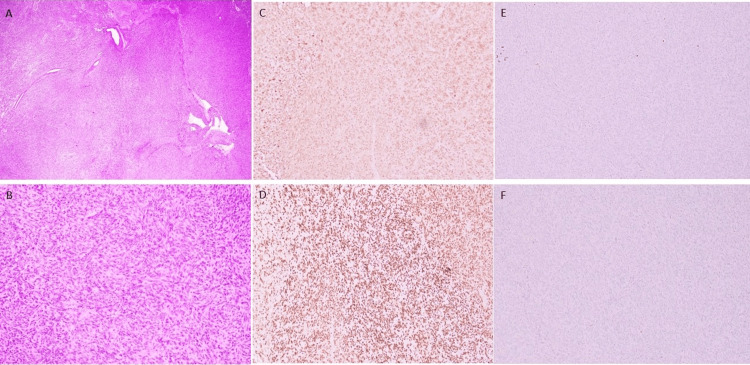
(A) Hematoxylin and eosin (H&E) (20X magnification) of the resection specimen shows a variegated appearance with alternating areas of high cellularity (Antoni A) and low cellularity (Antoni B). (B) H&E (200X magnification) shows fascicles of uniform spindle cells with fibrillary eosinophilic cytoplasm, the cells lack significant pleomorphism. (C-D) S100 and SOX10 IHC highlight positive Schwann cells, respectively. (E-F) The tumor cells are negative for HMB45 and NKX3.1, respectively.

Initially, the tumor board consensus was to observe the pelvic schwannoma. However, the patient reported progressively worsening urinary frequency and constipation by an extrinsic mass compression in the rectum, as shown in the colonoscopy. Consequently, two years after the diagnosis of metastatic prostate cancer, the tumor board decided that the patient would benefit from debulking surgery of the pelvic mass. An open resection of the pelvic mass was performed, revealing a tumor adherent to the bladder, prostate, and rectum. Histopathologic examination was consistent with schwannoma (Figure 3A-3B], as indicated by positive immunostains for S100 and SOX10 (Figure 3C-3D), while negative for cytokeratin AE1/AE3, melanocytic markers HMB45 and MelanA (Figure 3E). NKX3.1 and PSA were also negative; helping rule out prostate origin (Figure 3F). As per surgical oncology, there was no carcinomatosis in the peritoneum. However, due to capsular violation during the procedure, post-operative adjuvant therapy was recommended to control the risk of local progression of the pelvic schwannoma. A higher dose of radiation therapy will be applied for prostate cancer localized within the same vicinity for definite treatment. 

Regarding metastatic castrate-resistant prostate cancer, the patient is maintained on abiraterone/prednisone with ADT until present, which has thus far yielded an excellent biochemical response. The carcinomatosis has resolved, with no evidence of bone metastasis or gross progression of lymphadenopathy. 

## Discussion

Pelvic schwannoma

The WHO defines schwannoma as a nerve sheath tumor composed entirely or nearly entirely of differentiated neoplastic Schwann cells [3]. Schwannomas mostly occur as sporadic (90%) solitary lesions in the superficial soft tissue of the head, neck, and extremities [3]. Schwannomas in the pelvis are uncommon and represent 0.3-3.2% of schwannomas [4]. 

Pathology review 

Grossly, schwannomas can present with variable sizes; usually under 5 cm. However, pelvic schwannomas can grow larger in size before becoming symptomatic. The schwannoma reached 11 cm in the largest dimension in our case. They are surrounded by a true capsule composed of epineurium [3]. They have a tan-white to yellow cut surface, larger lesions can show areas of cystic change. 

Microscopically, they usually exhibit a characteristic marbled appearance of alternating areas of high and low cellularity called Antoni A and Antoni B, respectively. However, different morphologic variants which don’t seem to affect the clinical prognosis of the tumor exist. Our case represents ancient schwannoma; characteristic of long-standing lesions exhibiting degenerative changes. Cellular schwannomas are composed of predominantly Antoni A areas with variably increased mitotic activity [5]. Epithelioid schwannomas exhibit multilobulated growth of cells with eosinophilic cytoplasm and round nuclei arranged as single cells or nests, about 40% of cases have a loss of SMARCB1/INI1 expression [6]. Plexiform schwannomas are less common; they arise mainly in the skin and subcutaneous tissue and have a nodular growth pattern. Microcystic/reticular schwannoma is a rare entity that preferentially involves the viscera. 

Immunohistochemistry demonstrates strong and diffuse staining for S100 and SOX10, with variable staining of GFAP [3]. 

Although NF2-inactivating mutations are present in many sporadic schwannomas, it is not specific to this entity. No definite molecular profile for schwannoma is currently identified [3]. 

Diagnostic challenge

Due to the nature of the location of pelvic schwannomas, they tend to pose a diagnostic dilemma. They tend to reach very large sizes before presenting with significant symptoms. They frequently undergo degenerative changes like hemorrhage and cystic areas, making them challenging to diagnose by radiologic modalities [7]. They can be confused with more common lesions in that region as an abscess, adnexal mass, fibrosarcoma, or liposarcoma [7]. In fact, Zou et al. reported a case of intrapelvic schwannoma preoperatively misdiagnosed as a teratoma in a 61-year-old female [8]. Our patient had a co-existing stage 4 prostate cancer, which could have led to an inappropriate diagnosis of his pelvic mass without the tissue biopsy results. This emphasizes the vital role careful pathologic interpretation plays in accurate definitive diagnosis, and the importance of keeping schwannomas in the differential diagnosis of possible pelvic lesions. 

Long-standing schwannomas like in our case may have cystic changes with diffuse lymphocytic and histiocytic infiltration [9]. The use of immunohistochemical staining of identified cellular Antoni A areas maintains the typical strong positive S100 (cytoplasmic and nuclear) and SOX10 (nuclear) staining and negative HMB45 stains aid diagnosis of schwannoma and helps to rule out other differentials [5]. For our patient, the lack of NKX3.1 in the tumor cells also helped to confirm the diagnosis of schwannoma. 

## Conclusions

We present the first reported case of pelvic schwannoma presenting in synchrony with stage 4 prostate cancer. Schwannomas are slow-growing tumors; they can grow to a very large size in the pelvic region before causing symptoms. This can cause diagnostic confusion with more common entities or existing comorbidities in imaging studies due to the rarity of schwannoma in this location. Tissue diagnosis remains the gold standard for accurate final diagnosis. 
